# Presence Service in IMS

**DOI:** 10.1155/2013/606790

**Published:** 2013-07-23

**Authors:** David Petras, Ivan Baronak, Erik Chromy

**Affiliations:** Institute of Telecommunications, Faculty of Electrical Engineering and Information Technology, Slovak University of Technology in Bratislava, Ilkovičova 3, 812 19 Bratislava, Slovakia

## Abstract

This paper describes the presence service, which is located in the IP multimedia subsystem. This service allows making many applications for different groups of people. The paper describes differences between a network without the service and with the service. The biggest change is an increased number of transmitted messages. The presence uses some part of the IP multimedia subsystem control layer, which is shown in communication between the user and the server. The paper deals with the number of generated messages depending on the behaviour of the users. This is described by a mathematical model using discrete Markov chains.

## 1. Introduction

Originally, each service or group of services had its own network for its own use. The idea of the next generation networks (NGN) comes with the development of technology. This network would be shared by all services. IP multimedia subsystem (IMS) is an implementation of NGN [1–7]. IMS allows interworking between circuit switching and packet switching networks. IMS has many advantages. New services do not need to change the structure of the network. Quality of service is guaranteed through QoS parameters.

The presence service has two roles: to inform the user about the status of others and to inform others about the user's status [8]. It is transmitted through session initiation protocol for instant messaging and presence leveraging extension (SIMPLE) [9]. This protocol allows the transmission of messages over the network without changes, when the service is deployed. Messages are created as XML documents. These look like PIDF [10] and their extension like RPID [11].

Within the scope of the project “Support of Center of Excellence for SMART Technologies, Systems, and Services II” funded by structural funds from the European union, we have built the most modern IP multimedia subsystem lab at the Institute of Telecommunications. In this lab we can also conduct research aimed for services.

**Figure 1:**
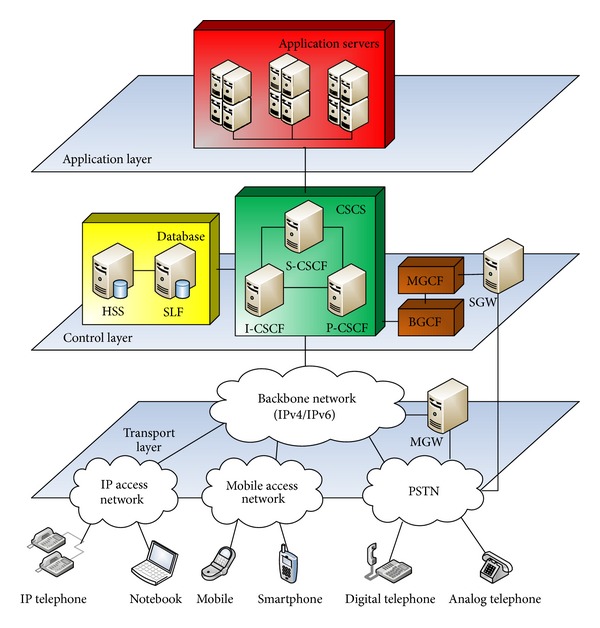
Architecture of IMS.

IMS consists of three layers: transport, control, and application (Figure 1). The transport layer is the lowest. This layer has two roles. First role is to secure an access of devices from different types of networks (GPRS, UMTS, IP, PSTN, etc.) through gateways like Media Gateway (MGW), Signaling Gateway (SGW). Second role is to transfer messages from the user to the control layer or from one user to another user through IP data network. The control layer is the core of the system. It controls the communication and creates connections between users. It directs messages through three call season control function (CSCF) servers. P-CSCF is an entry point to IMS. I-CSCF provides registration and interworking between two IMS. S-CSCF is a central point of direction. It communicates with the application servers. Home subscriber server (HSS) is a database where user profiles and data for the service are stored. The Subscriber location function (SLF) selects the database from several HSSs. The application layer provides services. There are three types of servers. SIP application server is used for applications using the SIP protocol. OSA application server is independent of the protocol through API between the server and the control layer. CAMEL application server is used for applications from legacy network [12].

## 2. Presence Architecture

**Figure 2:**
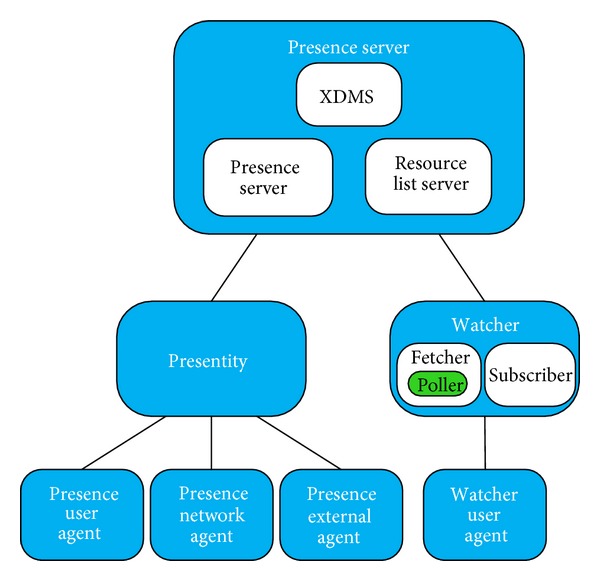
Presence architecture.

Presence architecture is shown in Figure 2. It has three levels: agents, entities, and a server. The agents collect information from various sources. The agents are various programmes. The presence user agent collects information from user devices. Presence network agent collects information from network elements. Presence external agent collects information from other networks. Watcher presence agent provides information to the watcher.

Entities are characterized by the fact that they can process the SIP messages (UE, S-CSCF, and AS). Entities are divided into two types. Presentity (presence entity) provides information about itself and the watcher observes the status of the others. Watchers are divided into three groups. The fetcher is only interested in the current status. Poller is a special kind of Fetcher, which observes status in certain time intervals. The subscriber also observes the changes in the presence of entities [13]. The server collects and sends information about users, which is stored in XML documents. The presence server receives messages and assigns it to the correct user. The resource list server creates lists of users for watchers and sends their status together. The XML document manager server (XDMS) supports other parts of the presence server. For example, XDMS knows that the watcher is authorized to observe the presence entity. Application server is designed so that it could control the number of messages. One of the possibilities is periodic sending of messages. If there are more messages than the server can send, it puts them into a waiting queue. If the waiting queue is full, then the server deletes the messages [14].

### 2.1. Communication

**Figure 3:**
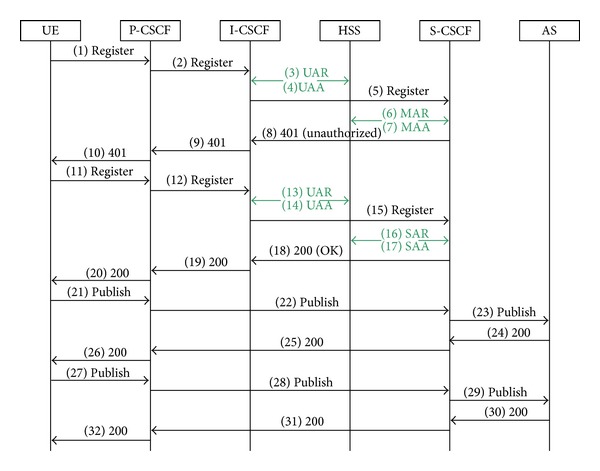
Publication status.

There are two processes of exchanging messages in the presence service. Process of publishing is shown in Figure 3. This exchange of messages has two parts. The first is registration (messages 1–20) and the second is publication of status (messages 21–32). S-CSCF is assigned to the user during registration. User equipment (UE) is a telephony device, which enters IMS through P-CSCF. P-CSCF through I-CSCF determines where to send the Register request. The information about where to send the message and about the user profile for S-CSCF is stored in the HSS. First, S-CSCF sends a response 401(unauthorized). After receiving answers, UE creates another Register request, after which the user will have successfully registered. A detailed description of the registration is in [15]. In messages 21–23 UE (presentity) sends its whole status in request Publish to the application server (presence server). Messages pass only through P-CSCF and S-CSCF after the registration. S-CSCF knows where the server is according to the initial filter a criteria (iFC). The filter is obtained from the HSS during the registration. Presence server sends confirmation message 200 (OK) as soon as possible, to prevent resending messages. When changing status, UE sends another request Publish, which will go the same way as the first one. The form of the messages is described in [16]. The message itself contains only the change of the status.

**Figure 4:**
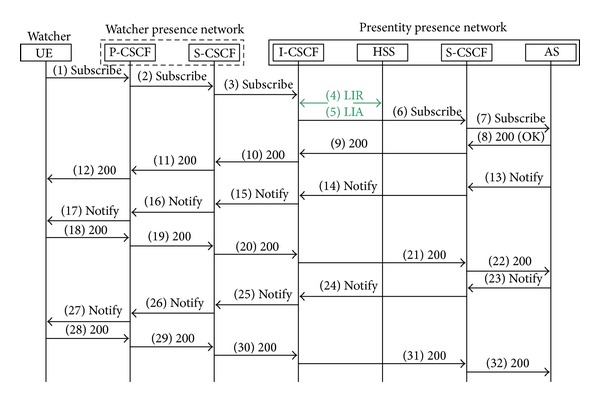
Subscribe status.

The process of subscribing is shown in Figure 4. The figure describes a situation, when the watcher is in another IMS network like presence server. Process of registration is the same as in the previous figure and therefore is not listed. Entry Point is I-CSCF to another IMS network. UE (watcher) creates request Subscribe. The filter is in the request [17]. In the filter, there is information about what the watcher wants to know. UE enters into its IMS network through P-CSCF. It continues to S-CSCF. S-CSCF sends subscribe from the watcher presence network to the presentity presence network. I-CSCF finds S-CSCF and S-CSCF sends request to AS, where there is a list of contacts with status. Upon receiving the request, the application server verifies the user's authority. If it is correct, the application server sends response 200 (OK). AS sends request Notify with the body where it contains the information about the presentity status. Type of watcher is Subscribe in Figure 4. If one of the presentity, which the watcher observes, changes its state, server sends another Notify message without request.

## 3. Deployment of the Service

Deployment of the presence service means three issues. Application server must be in the application layer. This server receives the information from agents, it stores the information to XML documents, and it sends the information to watcher according to the filter in the request. Agents must be added to the network. User agents are applications on the user's devices. Network agents are applications on network elements in the control layer (S-CSCF and HSS) and on servers in application layer (position server and application server of any other service). External agents collect information from other networks. Another important issue is the increased number of transmitted SIP messages. Fifty percent of transmitted messages are related to this service [18]. That is why it is most important to focus on the number of transmitted messages.

### 3.1. Messages

Presence service uses three types of SIP requests. publish is sent when presentity logs on (pub_login), logs off (pub_logout), modifies status (pub_modify), and refreshes status (pub_refresh) if it does not change the state. subscribe is sent when presentity starts (sub_initial), ends (sub_terminal), and refreshes (sub_refresh) to subscribe information from presence server. notify is sent by a server; that server notifies status of presentity to watchers (notify). These are eight situations, when someone sends a request [19]. The largest representation has request notify. Number of notify is given by (1). It is important to create a mechanism to control the number of sent Notify messages [20]. These messages occur after the server receives a message publish; hence it is important to focus on Publish messages:

(1) r_notify=Watchers·(r_pub_login+r_pub_logout  +r_pub_modify+r_pub_refresh).

### 3.2. Presence Server

**Figure 5:**
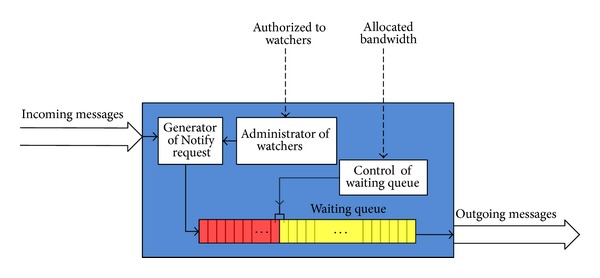
Logical scheme of the presence server.

In this paper, server creates Notify messages in Figure 5. Incoming messages are Publish requests. Generator of Notify request creates messages to send. Number of messages depends on incoming messages and the number of authorized watchers. Requests Notify are placed in the waiting queue. Messages are sent from the server periodically to avoid network congestion. It is assigned bandwidth for service. The bandwidth divided the waiting queue into two parts. Messages in yellow part of waiting queue are sent over time Δ*t*, and messages in the red part of waiting queue must wait for send in next period. If the waiting queue is full and other messages arrive, these massages will be deleted.

## 4. Model for Creation of Messages

**Figure 6:**
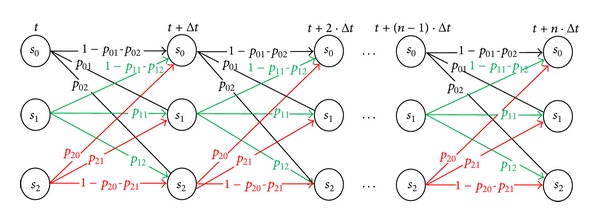
Model of making messages.

Creation of messages can be described by Markov chain shown in Figure 6. This model describes how many messages are created for time Δ*t* in dependency on the average time the users spend in individual states and their numbers. Users can be in three states. *s*_0_ represents online presentity that has unchanged status from time of its login or last change. *s*_1_ represents online presentity that changed its previous status. *s*_2_ represents offline presentity. Probability of transition from one state to another is given by exponential distribution [21] *p*_*ij*_ (*i* = 0, 1, 2; *j* = 0, 1, 2) as follows:

(2) $$\begin{array}{c} p_{ij}=\int_{0}^{\Delta t}\lambda_{ij}\cdot e^{-\lambda_{ij}x}dx, \end{array}$$

where *λ*_*ij*_ is given by the average time of creation message *t*_*ij*_,

(3) $$\begin{array}{c} \lambda_{ij}=\frac{1}{t_{ij}}. \end{array}$$

The probability, in which the user changes your state is given by the matrix:

(4) $$\begin{array}{c} \begin{array}{cccc} & s_{0} & s_{1} & s_{2}\\ s_{0} & 1-p_{01}-p_{02} & p_{01} & p_{02}\\ s_{1} & 1-p_{11}-p_{12} & p_{11} & p_{12}\\ s_{2} & p_{20} & p_{21} & 1-p_{20}-p_{21} \end{array}. \end{array}$$

States means the following:

 (i)
  (5) $$\begin{array}{c} P\left(s_{0},t\;|\;s_{0},\Delta t\right)=1-p_{01}-p_{02} \end{array}$$

is the probability in which online presentity does not change your presence status over time Δ*t*.

 (ii)
  (6) $$\begin{array}{c} P\left(s_{0},t\;|\;s_{1},\Delta t\right)=p_{01} \end{array}$$

is the probability in which online presentity changes your presence status over time Δ*t*.

 (iii)
  (7) $$\begin{array}{c} P\left(s_{0},t\;|\;s_{2},\Delta t\right)=p_{02} \end{array}$$

is the probability in which online presentity goes offline over time Δ*t*.

 (iv)
  (8) $$\begin{array}{c} P\left(s_{1},t\;|\;s_{0},\Delta t\right)=1-p_{11}-p_{12} \end{array}$$

is the probability in which online presentity does not change your presence status over time Δ*t*.

 (v)
  (9) $$\begin{array}{c} P\left(s_{1},t\;|\;s_{1},\Delta t\right)=p_{11} \end{array}$$

is the probability in which online presentity changes your presence status over time Δ*t*.

 (vi)
  (10) $$\begin{array}{c} P\left(s_{1},t\;|\;s_{2},\Delta t\right)=p_{12} \end{array}$$

is the probability in which online presentity goes offline over time Δ*t*.

 (vii)
  (11) $$\begin{array}{c} P\left(s_{2},t\;|\;s_{0},\Delta t\right)=p_{20} \end{array}$$

is the probability in which offline presentity goes online over time Δ*t*.

 (viii)
  (12) $$\begin{array}{c} P\left(s_{2},t\;|\;s_{1},\Delta t\right)=p_{21} \end{array}$$

is the probability in which offline presentity changes your presence status over time Δ*t*.

 (ix)
  (13) $$\begin{array}{c} P\left(s_{2},t\;|\;s_{2},\Delta t\right)=\;1-p_{20}-p_{21} \end{array}$$

is the Probability in which offline presentity stays offline over time Δ*t*.

States *s*_0_ and *s*_1_ have same probability, because it is a state when presentity is online. Dividing is only for the purpose of illustrating a different message. Offline presentity cannot go to state *s*_1_, because this state is created by the change of the online state. If we change parameters of publish modify and publish refresh, the number of other messages stays the same.

### 4.1. Meaning of Transition between States

Messages are created when someone goes from one state to another. The number of messages pub_modify is given by (14), the number of pub_login is given by (15), and the number of pub_logout is given by (16). Number of messages pub_refresh is counted differently. Probability of creation of messages is given by (17), where *R* is the time after which the user sends a message, if not, it changes its state, *t*_*m*_ is the average time of change presence status, and *t*_of_ is the average time of user log off. The number of messages pub_refresh is given by (18). The number of messages *s*_pub_*x* over interval 〈*T*_1_, *T*_2_〉 is given by (19), where *x* is the one has type of messages.

(14) pub_modify(t)=s0(t)·P(s0,t ∣ s1,t−Δt)+s1(t)·P(s1,t ∣ s1,t−Δt),

(15) $$\begin{array}{c} \text{pub}\_\text{login}\left(t\right)=s_{2}\left(t\right)\cdot P\left(s_{2},t\;|\;s_{0},t-\Delta t\right), \end{array}$$

(16) pub_logout(t)=s0(t)·P(s0,t ∣ s2,t−Δt) +s1(t)·P(s1,t ∣ s2,t−Δt),

(17) Pref=(1−∫0R1tm·e(−1/tm)xdx) +(1−∫0R1tof·e(−1/tof)xdx),

(18) $$\begin{array}{c} \text{pub}\_\text{refresh}\left(t\right)=s_{0}\left(t\right)\cdot P_{\text{ref}}+s_{1}\left(t\right)\cdot P_{\text{ref}}, \end{array}$$

(19) $$\begin{array}{c} s\_\text{pub}\_x=\int_{T_{1}}^{T_{2}}\text{pub}\_x\left(t\right)dt. \end{array}$$

The number of notify messages is given by the number of online watchers and the number of Publish messages as follows:

(20) notify(t) =watchers(t)·(pub_refresh(t)+pub_modify(t)+pub_login(t)+pub_logout(t)).

## 5. Using the Model

A network with 550 000 users is given. They are assigned into online users in states *s*_0_ and *s*_1_ and offline users in the state *s*_2_. *t*_*on*⁡_ is the average time of the user being in an online state. *t*_*off*⁡_ is the average time of the user being in the offline state.

If *t*_*on*⁡_ is more than *t*_*off*⁡_, then the number of online users increases. The number of online users decreases in another case. *t*_*m*_ is the average time of change of user state. Time Δ*t* represents time, when server collects information and subsequently sends them periodically.

**Figure 7:**
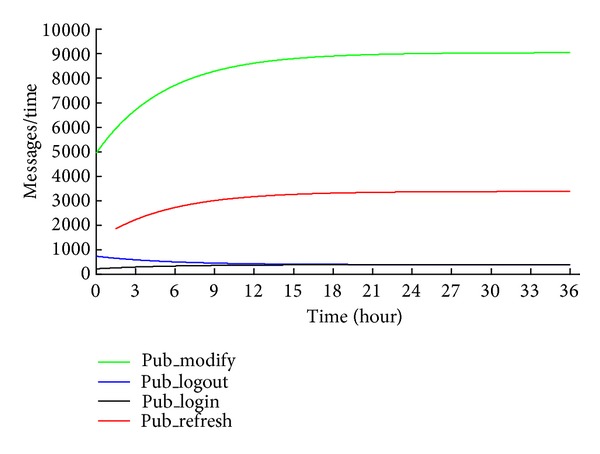
Messages in system A.

System A describes a situation, where the number of online users increases with time. The number of messages created over Δ*t* is shown in Figure 7. The system is characterized by the following:

 
*s*_0_ = 150 000,
 
*s*_1_ = 50 000,
 
*s*_2_ = 350 000,
 
*t*_*on*⁡  _ = 480 minutes,
 
*t*_*off*⁡_ = 240 minutes,
 
*t*_*m*_ = 20 minutes,
 Δ*t* = 0.5 minutes,
 
*R* = 45 minutes.

**Figure 8:**
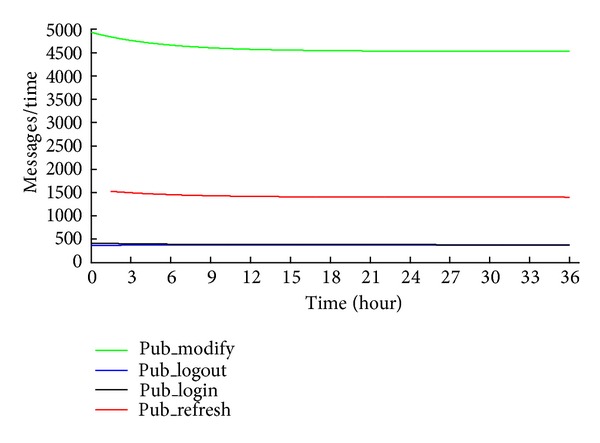
Messages in system B.

System B describes a situation, where the number of online users decreases over time. The number of messages is shown in Figure 8. The system is characterized by the following:

 
*s*_0_ = 150 000,
 
*s*_1_ = 50 000,
 
*s*_2_ = 350 000,
 
*t*_*on*⁡_ = 240 minutes,
 
*t*_*off*⁡_ = 480 minutes,
 
*t*_*m*_ = 20 minutes,
 Δ*t* = 5 minutes,
 
*R* = 45 minutes.

**Figure 9:**
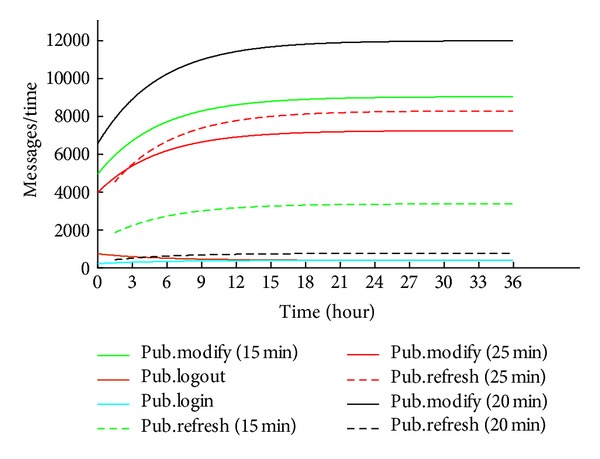
System A with increasing amount of messages.

Figure 9 shows the number of messages pub_modify at different average times of change status *t*_*m*_. Decrease in *t*_*m*_ would mean adding more applications to the network. If changes are more frequent, the amount of messages is increased.

**Figure 10:**
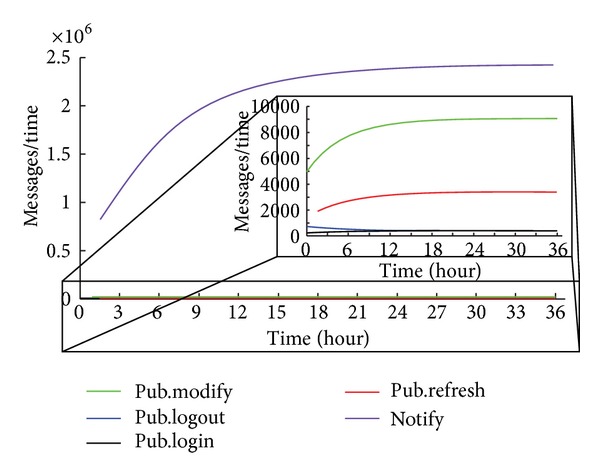
System A with Notify messages.

Figure 10 shows the number of Notify messages, where the number of watchers of one's presentity is increasing with the number of online users. The number of messages is calculated by (19), where the number of watchers is given by (21). *k* represents the ratio of the amount of users in the telephone list and the amount of all users.

One has

(21) $$\begin{array}{c} \text{watchers}\left(t\right)=\left(s_{0}+s_{1}\right)*k. \end{array}$$

For Figure 10, *k* = 0.005.

## 6. Conclusion

Presence service is one of the key services in IMS. It allows the creation of a huge amount of applications, which can share information. Protection of this information is important. We can find some information about protection in [22, 23]. We have to consider some aspects before its deployment. The service requires creation of agents on the user's devices and some blocks of IMS. Agents collect information and send it to the presence server. The presence server must be at the application layer in which the server processes incoming messages and sends information about the user's state. It is necessary to create a mechanism that controls the number of transmitted messages, because a huge amount of messages can overload the network. We need to determine the number of messages transmitted by the network before designing this mechanism. The model described in this paper can be used for this. This model displays the number of incoming and outgoing messages from the network. Users are in various states at the beginning, and gradually they log out, log in, and change their presence status. The change of the number of transmitted messages is related to this. If we observe this long enough without changing probability, the model will be in a stable state. That means that the same number of users changes their state in every step. We can determine the expected number of messages from the model, and according to this, we can design the size of the waiting queue on the presence server. We can see the ratio of messages if we want to assign different priorities too.
